# Bilateral Wrist Septic Arthritis Caused by Group B β-Hemolytic Streptococcus: A Case Report

**DOI:** 10.7759/cureus.110067

**Published:** 2026-06-01

**Authors:** Adam C Dickerson, Mebeli Becerra, Chinmay Patel, Eric H Chou

**Affiliations:** 1 Emergency Medicine, Baylor Scott and White All Saints Medical Center, Fort Worth, USA; 2 Emergency Medicine, Burnett School of Medicine at Texas Christian University (TCU), Fort Worth, USA; 3 Emergency Medicine, Taipei Hospital, Ministry of Health and Welfare, New Taipei City, TWN

**Keywords:** group b streptococcus, immunocompromised hosts, miscarriage, post-liver transplantation, septic arthritis

## Abstract

Septic arthritis is typically a monoarticular infection most commonly caused by *Staphylococcus aureus*; however, polyarticular presentations are uncommon and associated with higher morbidity and mortality. Group B β-hemolytic Streptococcus is a less frequent pathogen but may occur in patients with significant comorbidities or recent obstetric events. Early recognition is critical, as delayed diagnosis may result in joint destruction and systemic complications. We report a case of a 40-year-old woman with a history of autoimmune hepatitis status post liver transplantation on chronic immunosuppression, rheumatoid arthritis, and recent miscarriage with retained products of conception, who presented with bilateral wrist pain and swelling, worse on the left. Initial evaluation suggested an inflammatory arthritis flare due to polyarticular involvement. However, laboratory studies revealed leukocytosis and elevated inflammatory markers. Arthrocentesis of the left wrist was performed, and cultures grew group B β-hemolytic *Streptococcus*. The patient subsequently underwent bilateral dorsal wrist arthrotomies with synovectomy, irrigation, and debridement, yielding purulent fluid from both joints. Blood cultures confirmed bacteremia with the same organism. She was treated with intravenous ceftriaxone for a planned four-week course, with clinical improvement. Evaluation for endocarditis was negative. This case highlights a rare presentation of polyarticular septic arthritis involving bilateral wrists caused by group B β-hemolytic *Streptococcus* in an immunocompromised patient following miscarriage. Clinicians should maintain a high index of suspicion for septic arthritis in patients with multiple risk factors, even when presentation mimics inflammatory arthritis, as prompt diagnosis and intervention are essential to improving outcomes.

## Introduction

Septic arthritis is an infection of a joint caused by bacteria or other microorganisms. Its reported incidence of 1-35 cases per 100,000 persons worldwide and 4-10 cases per 100,000 persons in the United States (US) [1,2]. In 2012, septic arthritis accounted for approximately 16,000 emergency department (ED) visits in the US and was associated with an estimated mortality rate of 5-15% [1-3]. Several predisposing factors have been associated with septic arthritis, including skin infections, intra-articular injections, prosthetic joints, recent joint surgery, diabetes mellitus, human immunodeficiency virus (HIV) infection, intravenous drug use, osteoarthritis (OA), rheumatoid arthritis (RA), gonorrhea, immunosuppression, age greater than 80 years, and smoking [1-3]. Patients with RA are at particularly high risk because chronic synovial inflammation can lead to joint damage, while immunosuppressive therapy further increases susceptibility to bacterial colonization and infection [1]. Prior studies have reported an incidence of septic arthritis of 1.8 per 1,000 patient-years among patients treated with non-biologic disease-modifying anti-rheumatic drugs (DMARDs), compared with 4.2 per 1,000 patient-years among those receiving anti-tumor necrosis factor (TNF) therapy [1].

Septic arthritis involving a single joint accounts for approximately 80-90% of all cases. *Staphylococcus aureus* is the most common causative organism, and the knee is the most frequently affected joint [4,5]. The overall mortality rate is estimated to be approximately 9% [4,5]. Although less common, group B β-hemolytic *Streptococcus* species should also be considered as potential pathogens, particularly in the setting of peripartum bacteremia associated with retained products of conception [4,5]. Polyarticular septic arthritis is a less frequent presentation but is associated with significantly worse outcomes, with an estimated mortality rate of 23% [5-7]. In cases caused by group B β-hemolytic *Streptococcus*, the incidence of multiple-joint involvement increases to approximately 32% [5-7]. When occurring in patients with RA, mortality may rise to as high as 56% [6,7]. Progression to septic shock further increases mortality, reaching up to 69% [1,3,6,7].

In this report, we present a rare case of bilateral wrist septic arthritis caused by group B β-hemolytic *Streptococcus* in a patient with a history of RA, liver transplantation, and a recent miscarriage complicated by retained products of conception. She presented with bilateral wrist pain and edema, more pronounced on the left, accompanied by warmth, mild erythema, and minimal limitation in range of motion. This case underscores the importance of maintaining a high index of suspicion for septic arthritis and a low threshold for initiating empiric antibiotic therapy in patients with multiple risk factors for polyarticular involvement.

## Case presentation

A 40-year-old woman with a medical history significant for autoimmune hepatitis status post liver transplantation 10 years ago, maintained on chronic immunosuppressive therapy, hypertension, and RA treated with prednisone 40 mg daily, presented to the ED with concern for a possible RA flare. She reported chronic polyarthritis involving both wrists, hands, and knees over the preceding two to three months, with acute worsening of left wrist pain beginning two days prior to presentation. She denied any recent trauma or injury. Although she reported a subjective fever at home, she remained afebrile thereafter. Of note, the patient had presented to the ED five days earlier with heavy vaginal bleeding and cramping and was diagnosed with a miscarriage. Pelvic examination at that time revealed an open cervical os (*os uteri*), and ultrasonography confirmed a failed intrauterine pregnancy with retained products of conception and associated hemorrhage.

On initial ED evaluation, vital signs were within normal limits: blood pressure 121/73 mmHg, heart rate 79 beats per minute, temperature 98.6 °F, respiratory rate 16 breaths per minute, and oxygen saturation 100% on room air. Laboratory evaluation demonstrated leukocytosis, with a white blood cell count of 18,400/mm³, and elevated inflammatory markers, including an erythrocyte sedimentation rate (ESR) of 72 mm/hour and a C-reactive protein (CRP) level of 13.2 mg/dL. Liver biochemical studies were also elevated, including an alkaline phosphatase (ALP) level of 395 U/L, aspartate aminotransferase (AST) of 89 U/L, and alanine aminotransferase (ALT) of 69 U/L, findings consistent with her underlying autoimmune hepatitis and cirrhosis (Table 1).

**Table 1:** Initial laboratory findings on presentation Laboratory findings on initial presentation demonstrating leukocytosis, markedly elevated inflammatory markers, and abnormal liver function tests consistent with underlying chronic liver disease and systemic inflammation.

Laboratory Tests	Patient Results	Normal Range
White blood cell count	18,400/mm³	4500-11,000/mm³
Erythrocyte sedimentation rate	72 mm/hour	0-20 mm/hour
C-reactive protein	13.2 mg/dL	<0.5 mg/dL
Alkaline phosphatase	395 U/L	25-100 U/L
Aspartate aminotransferase	89 U/L	12-38 U/L
Alanine aminotransferase	69 U/L	10-40 U/L

**Figure 1:**
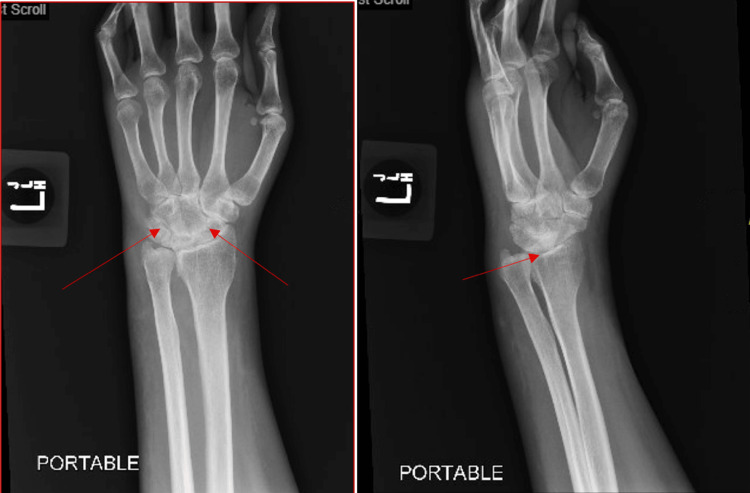
Radiograph of the left wrist demonstrating arthropathic changes with joint space narrowing involving the carpal articulations. Chondrocalcinosis of the radiocarpal joint is present, along with mild diffuse soft tissue swelling.

Physical examination revealed a swollen, warm, and tender left wrist with minimal limitation in range of motion. These findings raised concern for both a flare of RA and septic arthritis, particularly in the context of her recent miscarriage with retained products of conception. A radiograph of the left wrist was obtained (Figure 1), followed by venous Doppler ultrasonography of the left upper extremity, which was negative for deep venous thrombosis. Arthrocentesis of the left wrist was performed in the ED, and synovial fluid was sent for analysis. Orthopedic surgery was subsequently consulted.

The following day, the patient was taken to the operating room for bilateral dorsal wrist arthrotomies with synovectomy, irrigation, and debridement. Intraoperatively, approximately 1-2 mL of gross purulent fluid was drained from each wrist joint. Both joints demonstrated hyperemic, inflamed synovial tissue, with the left wrist more severely affected than the right. Prior to capsular closure, 500 mg of vancomycin powder was instilled into each joint space. Synovial fluid and blood cultures subsequently grew group B β-hemolytic *Streptococcus* (Table 2).

**Table 2:** Microbiologic findings from synovial fluid and blood cultures Synovial fluid and blood culture results obtained during initial evaluation and hospitalization. Synovial fluid analysis demonstrated a marked inflammatory response with numerous white blood cells and the absence of crystals. Cultures from both synovial fluid and blood grew group B β-hemolytic *Streptococcus agalactiae*, confirming the diagnosis of bacteremia with associated septic arthritis. Gram stain of blood cultures revealed gram-positive cocci in pairs and chains, consistent with streptococcal species. Both isolates were reported as uniformly susceptible to penicillin.

Laboratory Tests	Patient Results	Normal Range
Synovial Fluid Gram Stain	Many white blood cells. No organisms seen	Very few to no white blood cells, no pathogenic organisms
Synovial Fluid Culture, Body Fluid	β-hemolytic *Streptococcus*, Group B, Species uniformly penicillin susceptible	No pathogenic organisms
Synovial Fluid Crystals	Absent	Negative
Blood Culture Gram Stain	Gram-positive cocci in pairs and chains (2 of 2 sets positive)	No pathogenic organisms
Culture, Blood	β-hemolytic *Streptococcus*, Group B, Species uniformly penicillin susceptible	No pathogenic organisms

The patient was diagnosed with group B β-hemolytic *Streptococcus* bacteremia complicated by bilateral septic arthritis of the wrists. In accordance with recommendations from the infectious disease service, she was initiated on intravenous ceftriaxone at a dose of 2 g daily, with a planned treatment duration of four weeks. A transthoracic echocardiogram (TTE) was obtained to evaluate for infective endocarditis and demonstrated no evidence of valvular vegetations. Repeat blood cultures showed no further growth.

## Discussion

We present a rare case of bilateral wrist septic arthritis caused by group B β-hemolytic *Streptococcus* in a 40-year-old woman with a history of autoimmune hepatitis, cirrhosis, hypertension, RA, and recent miscarriage. Group B β-hemolytic *Streptococcus* is a gram-positive organism that commonly colonizes the lower genitourinary and gastrointestinal tracts and is well recognized as a leading cause of neonatal sepsis [7-9]. Although historically associated with the intrapartum period, infections are increasingly reported in adults, particularly among patients with comorbidities such as diabetes mellitus, immunodeficiency, chronic liver disease, malignancy, and miscarriage. Our patient presented to the ED five days prior with miscarriage and retained products of conception, which were removed at bedside. It remains unclear whether follow-up imaging was performed to confirm complete evacuation. These factors likely contributed to an increased risk of group B β-hemolytic *Streptococcus* infection and subsequent hematogenous dissemination, ultimately leading to polyarticular septic arthritis. Immunosuppression is a well-established risk factor for invasive group B β-hemolytic *Streptococcus* infection, with prior studies demonstrating associations with neutropenia and glucocorticoid use [10]. In this case, chronic prednisone therapy, combined with a history of liver transplantation and autoimmune disease, further increased susceptibility to opportunistic infection. 

Septic arthritis most commonly involves large joints, particularly the knee, whereas wrist involvement and polyarticular disease are relatively uncommon [1-3]. Early recognition and treatment are critical, as delayed intervention may result in rapid joint destruction, prolonged hospitalization, and increased mortality [2,3]. Management includes prompt arthrocentesis with synovial fluid analysis, early initiation of empiric antibiotic therapy, and surgical drainage when indicated [11,12]. In this case, the patient underwent arthrocentesis in the ED and was started on intravenous antibiotics within 24 hours. However, the synovial fluid specimen clotted, precluding cell count analysis. This is clinically significant, as a synovial white blood cell count >50,000/µL with neutrophil predominance is often used to support the diagnosis and guide management decisions. Despite the absence of this data, the patient underwent urgent bilateral wrist irrigation and debridement with evacuation of purulent material prior to the availability of culture results. Blood cultures obtained before antibiotic administration subsequently grew group B β-hemolytic *Streptococcus*, highlighting the importance of early culture acquisition to preserve diagnostic yield [1,2,7]. Based on infectious disease recommendations, the patient completed a four-week course of intravenous ceftriaxone via a peripherally inserted central catheter (PICC). TTE and repeat blood cultures excluded infective endocarditis and persistent bacteremia prior to discharge. 

Polyarticular septic arthritis is most commonly associated with hematogenous spread, particularly in patients with RA, and is often caused by organisms such as *Escherichia coli*, *Pseudomonas aeruginosa*, and group B β-hemolytic *Streptococcus* [1-3]. In this case, the most likely source of infection was hematogenous dissemination from retained products of conception following miscarriage. This case underscores the importance of maintaining a high index of suspicion for septic arthritis in immunocompromised patients presenting with acute joint symptoms. Given the potential for rapid progression to systemic complications, timely diagnosis, early antibiotic therapy, and appropriate surgical intervention are essential to improving outcomes. Our patient’s prompt evaluation and management likely prevented progression to septic shock [13]. Functional recovery was assessed using the Activity Measure for Post-Acute Care (AM-PAC) score [14], which improved from 12 on admission to 14 at discharge, indicating partial recovery without the need for inpatient rehabilitation. At follow-up, the patient reported improvement in acute symptoms, with residual limitations attributed to her baseline RA rather than sequelae of septic arthritis. 

## Conclusions

This case highlights the diagnostic challenge of septic arthritis in immunocompromised patients, particularly when presenting with polyarticular involvement. Clinicians should maintain a high index of suspicion for infection in patients with risk factors such as RA, immunosuppression, or recent obstetric or gynecologic events, as delayed treatment is associated with worse outcomes. Prompt recognition, early arthrocentesis, and timely initiation of antibiotic therapy are critical to preventing joint destruction and systemic complications.
